# Micafungin Inhibits Dengue Virus Infection through the Disruption of Virus Binding, Entry, and Stability

**DOI:** 10.3390/ph14040338

**Published:** 2021-04-07

**Authors:** Yen-Chen Chen, Jeng-Wei Lu, Chia-Tsui Yeh, Te-Yu Lin, Feng-Cheng Liu, Yi-Jung Ho

**Affiliations:** 1 School of Pharmacy, National Defense Medical Center (NDMC), No. 161, Section 6, Minquan East Road, Taipei 114, Taiwan; a0979288581@gmail.com; 2 Department of Biological Sciences, National University of Singapore (NUS), 14 Science Drive 4, Singapore 117543, Singapore; jengweilu@gmail.com; 3 School of Life Science, National Taiwan Normal University (NTNU), No. 88, Ting-Chow Road, Section 4, Taipei 116, Taiwan; emerald0620@gmail.com; 4 Institute of Preventive Medicine, National Defense Medical Center (NDMC), No. 172, Dapu Road, New Taipei City 237, Taiwan; 5 Division of Infectious Diseases and Tropical Medicine, Department of Internal Medicine, Tri-Service General Hospital (TSGH), National Defense Medical Center (NDMC), No. 161, Section 6, Minquan East Road, Taipei 114, Taiwan; lin.deyu@msa.hinet.net; 6 Rheumatology/Immunology and Allergy, Department of Internal Medicine, Tri-Service General Hospital (TSGH), National Defense Medical Center (NDMC), No. 161, Section 6, Minquan East Road, Taipei 114, Taiwan; lfc10399@yahoo.com.tw; 7 Graduate Institute of Life Sciences, National Defense Medical Center (NDMC), No. 161, Section 6, Minquan East Road, Taipei 114, Taiwan

**Keywords:** antiviral, dengue virus, micafungin, echinocandins, binding, entry and virucidal

## Abstract

Dengue fever is an arbovirus disease caused by infection with the dengue virus (DENV). Half of the world’s population lives under the threat of dengue fever, however, researchers have yet to develop any drugs that are clinically applicable to this infection. Micafungin is a member of the echinocandins family of anti-fungal drugs, capable of blocking the synthesis of β-1,3-D-glucan in the walls of fungal cells. Previous studies have demonstrated the effectiveness of Micafungin against infections of enterovirus 71 (EV71) and chikungunya virus (CHIKV). This is the first study demonstrating the effectiveness of micafungin in inhibiting the cytopathic effects of dengue virus serotype 2 (DENV-2) in a dose-dependent manner. Time-of-addition assays verified the inhibitory effects of micafungin in pre-treated, co-treated, and full-treatment groups. Binding and entry assays also demonstrated the effectiveness of micafungin in the early stage of DENV-2 infection. The virucidal efficacy of micafungin appears to lie in its ability to destroy the virion. Molecular docking assays revealed the binding of micafungin to the envelope protein of DENV-2, thereby revealing the mechanism by which micafungin affects the early stage of DENV infection and the stability of DENV. Two other micafungin analogs, caspofungin and anidulafungin, were also shown to have the antiviral effects on DENV-2. Finally, immunofluorescence assay (IFA) and reverse-transcription quantitative polymerase chain reaction (RT-qPCR) confirmed the broad anti-DENV ability of micafungin against dengue virus serotypes 1, 3, and 4 (DENV-1, DENV-3, and DENV-4). Taken together, these results demonstrate the potential of micafungin and its analogs as candidates for the development of broad-spectrum treatments for DENV infection.

## 1. Introduction

The dengue virus (DENV) is endemic to tropical and subtropical areas, putting nearly half of the world’s population at risk [1]. The incidence of dengue infection has increased by an estimated 30-fold in recent decades [2,3]. It has been estimated that annually, 390 million people are infected by DENV, resulting in 96 million cases of dengue fever [1]. The symptoms of dengue virus infection vary widely, from asymptomatic illness to severe dengue hemorrhagic fever/dengue shock syndrome (DHF/DSS). Dengue fever is characterized by acute-onset fever with various other manifestations, including severe headache, rash, retro-orbital pain, myalgias, and arthralgias [3,4]. DHF/DSS frequently occurs in cases of secondary infection with heterotypic dengue virus, which tends to mediate the symptoms via antibody enhancement [5].

DENV is a single-stranded, positive-sensed RNA virus classified within the *Flaviviridae* family with four serotypes. In a mature virion, E protein dimers and membrane proteins are arranged on the surface, which is a lipid bilayer derived from the membrane of host cells [6]. Beneath the surface, genomic RNA wrapped within the capsid protein encodes three structural proteins and seven non-structural proteins. The envelope protein is critical to viral attachment and fusion. Binding of the envelope protein to the host receptor triggers clathrin-mediated endocytosis causing DENV to enter into the endosome. The acidic environment in the endosome triggers the rearrangement of envelope proteins from dimer to trimer structures, resulting in fusion with the endosomal membrane and the release of viral genomic RNA into the cytoplasm of the host cell [7,8].

At present, some Food and Drug Administration (FDA)-approved drugs have found the anti-DENV effects, such as eugeniin from clove [9]. The micafungin is also an FDA-approved drug, which has been approved for the prevention and treatment of candida infection [10]. Recent studies have reported that micafungin also has antiviral activity toward enterovirus 71 (EV71) and chikungunya virus (CHIKV) [11,12]. At present, the effect of micafungin in cases of DENV infection remains unclear. Our primary objective in this study was to determine whether micafungin inhibits DENV infection and if so, to elucidate the underlying mechanism(s).

## 2. Results

### 2.1. Micafungin Presented Anti-DENV-2 Ability

**Figure 1:**
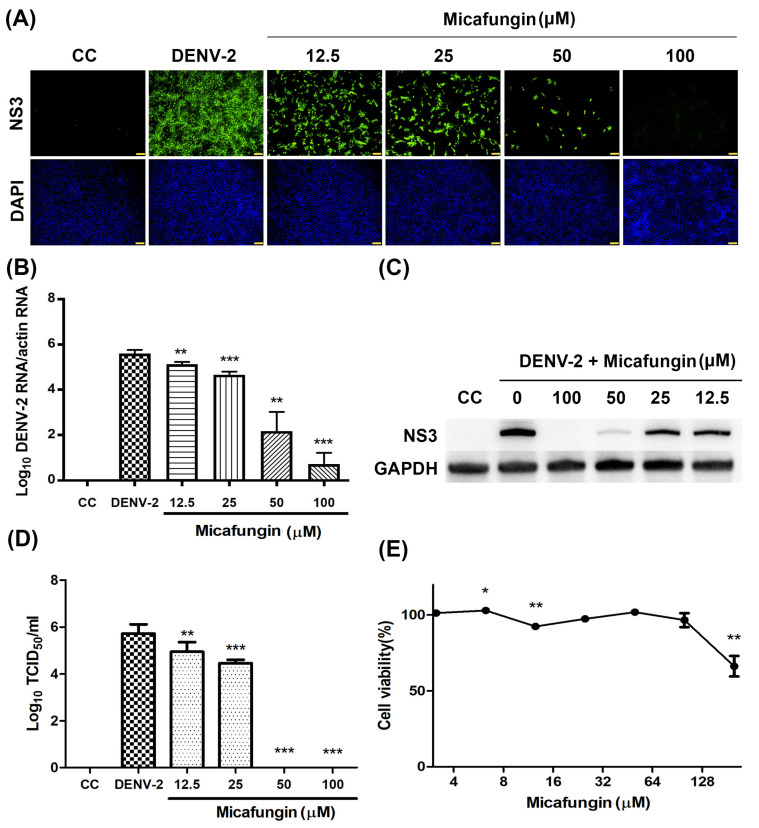
Anti-dengue virus serotype 2 (DENV-2) activity and cytotoxicity of micafungin in Vero cells: (**A**) DENV NS3 antibodies (upper) determined DENV-2 infection and DAPI (below) determined the nuclear by IFA; (**B**) RT-qPCR assay results of DENV-2 RNA; (**C**) Protein expression of dengue NS3 and GAPDH, as detected by Western blot analysis; (**D**) TCID_50_ assay results of DENV-2 yield; (**E**) Cytotoxicity of micafungin following treatment for a period of 3 days, as measured using cell viability assay. All data were obtained from at least three independent experiments, where significance was indicated as follows: * *p* < 0.05; ** *p* < 0.01; *** *p* < 0.001. Scale bar: 100 μM.

The anti-DENV activity of micafungin was first verified by IFA (Figure 1A). Vero cells infected with DENV-2 at multiplicity of infection (MOI) = 1 and inoculated with micafungin at concentrations of 12.5–100 μM were seeded in 48-well plates and incubated for 2 days. Micafungin was shown to significantly inhibit DENV infection in a dose-dependent manner, with the most pronounced activity occurring at a concentration of 100 μM. Quantitative reverse transcription PCR (RT-qPCR), western blot analysis, and 50% cell culture infectious dose (TCID_50_) assays were also used to investigate the inhibition activity of micafungin. Vero cells infected with DENV-2 at MOI = 0.1 and inoculated with micafungin at concentrations of 12.5–100 μM were seeded in 24-well plates. After 1 h absorption, the inoculum was replaced with fresh medium containing micafungin at indicated concentrations and incubated for 3 days. The data in Figure 1B shows that micafungin (12.5–100 μM) significantly reduced viral RNA levels, with a 4.86-log reduction at a concentration of 100 μM. At concentrations of 12.5–100 μM, micafungin was shown to decrease NS3 protein expression without affecting the GAPDH expression (Figure 1C). Micafungin also significantly reduced viral progeny yield, particularly at concentrations of 50 or 100 μM (Figure 1D). Cell viability assays revealed that the micafungin had significant cytotoxic effects at a concentration of 200 μM. The concentrations in the current study (6.25–100 μM) were safe to treat (Figure 1E).

### 2.2. Micafungin Inhibition Effects in the Early Stage of DENV-2 Infection

**Figure 2:**
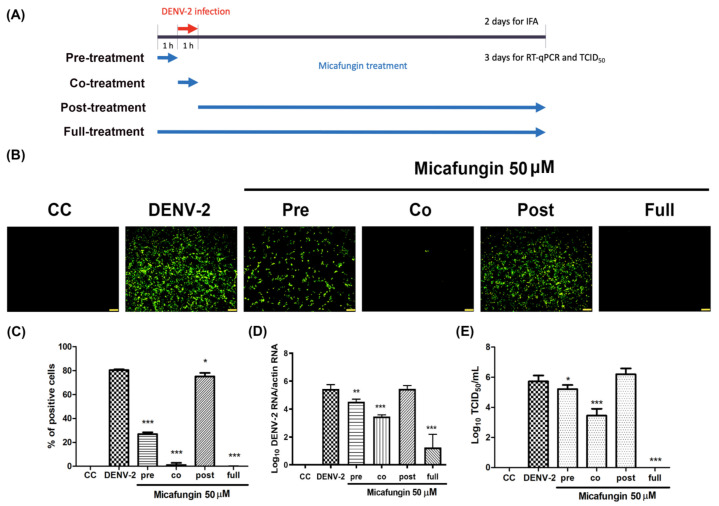
Time-of-addition assay of micafungin: (**A**) timeline of time-of-addition assay; (**B**) IFA results indicating DENV-2 infection; (**C**) quantitative analysis using ImageJ software; RNA level (**D**) and viral yield (**E**), as detected using RT-qPCR and TCID_50_ assays. All data were obtained from at least three independent experiments, where significance was indicated as follows: * *p* < 0.05; ** *p* < 0.01; *** *p* < 0.001. Scale bar: 100 μM.

Time-of-addition assays were used to identify the stages in which micafungin was effective. Vero cells were infected with DENV-2 at MOI = 1 for 1 h absorption, whereupon the inoculum was replaced with fresh medium followed by incubation for 2 days. Micafungin (50 μM) was added at four time points: 1 h prior to virus absorption (pre-treatment), during virus absorption (co-treatment), after virus absorption (post-treatment), and during all three of the above-mentioned periods (full-treatment) (Figure 2A). The result of IFA (Figure 2B) and corresponding quantitative analysis (Figure 2C) revealed that micafungin was most effective under full-treatment and co-treatment conditions with partial effects observed under pre-treatment conditions. A minor inhibition was observed under post-treatment. Viral RNA levels and progeny yields were verified by RT-qPCR and TCID_50_ assays following incubation at MOI = 0.1 for 3 days. Micafungin reduced the viral RNA levels by nearly 4-log under full-treatment conditions and 2-log under co-treatment conditions (Figure 2D). Micafungin significantly inhibited progeny yield under full-treatment (nearly 6-log reduction) and co-treatment conditions (2-log reduction) (Figure 2E). Partial effects were observed under pretreatment conditions, but no effects were observed under post-treatment conditions. The results of time-of-addition assays revealed that micafungin acts mainly during the early stages of DENV-2 infection.

### 2.3. Micafungin Might Bind into DENV-2 Envelope Proteins to Affect Viral Binding and Also Possess the Virucidal Ability

**Figure 3:**
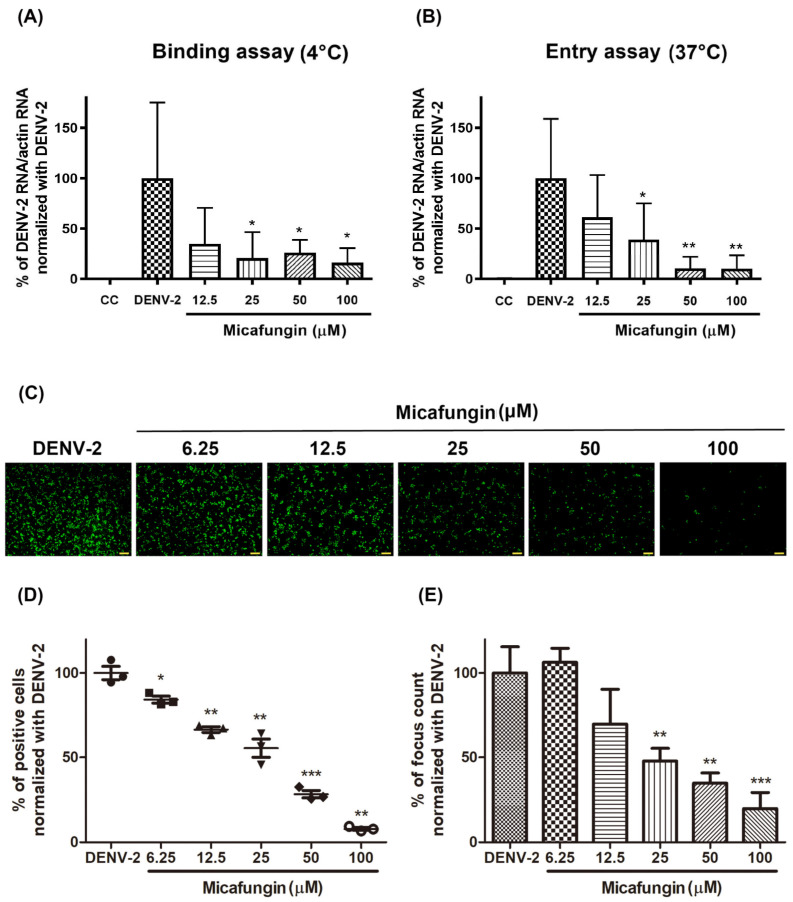
(**A**) Binding assay of micafungin; (**B**) entry assay of micafungin. RT-qPCR was used to quantify the amount of virus binding and entry. Each group was normalized with actin RNA expression and further compared to the DENV-2 group. (**C**) Virucidal assays of micafungin. IFA was used to estimate the infectious ability of DENV-2. Infection rate was determined using (**D**) ImageJ software and (**E**) foci counting. Data was normalized with the DENV-2 group to obtain the percentage of infection. All data were obtained from at least three independent experiments, where significance was indicated as follows: * *p* < 0.05; ** *p* < 0.01; *** *p* < 0.001. Scale bar: 100 μM.

**Figure 4:**
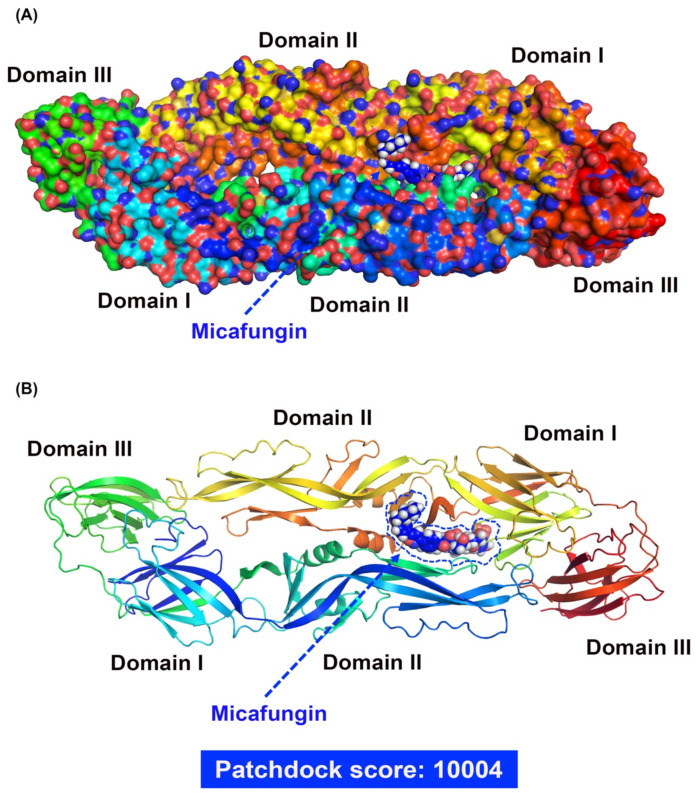
(**A**) Spheres rendered overview of DENV-2 envelope protein docked with micafungin and (**B**) interaction of DENV-2 envelope proteins with micafungin via molecular docking analysis.

Early-stage DENV-2 infection was characterized by viral binding to the receptor on the cell surface and entry into the cytoplasm via endocytosis. During the infection stage, the cells were maintained at a low temperature (4 °C), which resulted in the virus bound only onto the host receptor without entering the cytoplasm [13]. Thus, Vero cells were infected with DENV-2 (MOI = 10 at 4 °C for binding assay, and at 37 °C for entry assay) and treated with micafungin at indicated concentrations for 2 h. The inoculum was removed and the cells were washed by phosphate buffered saline (PBS) twice. The RNA of treated cells was extracted immediately and the level of viral binding and viral entry was determined by RT-qPCR. Micafungin appeared to inhibit DENV-2 binding and entry, reaching statistical significance at the dosages of 12.5–100 μM (Figure 3A,B). The inhibition trends of viral binding and entry were similar which implied that micafungin mainly inhibited viral binding effect during viral entry processing. Virucidal assays were used to assess the direct influence of micafungin on DENV-2 following incubation at indicated concentrations at 37 °C for 1 h. All groups were diluted 100-fold to eliminate micafungin residue, whereupon the dilutions were added to Vero cells in 24-well plates. Following incubation at 37 °C for 1 h, the supernatant was removed and the cells were covered with Dulbecco’s Modified Eagle Medium (DMEM) containing 1.5% (Weight/Volume; *w*/*v*) methylcellulose and 2% FBS for a period of 2 days. IFA was used to reveal the outcome (Figure 3C). Micafungin was shown to reduce the foci of DENV-2 in a dose-dependent manner. The percent of infected cells (Image J software; Figure 3D) and foci forming unit count (Figure 3E) also revealed that micafungin significantly reduced the pathogenicity of DENV-2. Molecular docking results revealed micafungin embedded into the cavity formed by domains 1 and 2 of the DENV-2 envelope protein, with a high score of 10,004 (Figure 4A,B) by Patchdock. Based on the predicted data of Patchdock, we labeled the hydrogen bond in blue and the covalent bond in orange. Micafungin possessed the hydrogen bonds and the covalent bonds in the prediction. Anidulafungin and caspofungin only possessed hydrogen bonds. The main structure of cyclic hexapeptides seems to be the main action site (Supplementary Table S2).

### 2.4. Anti-DENV-2 Activity of Micafungin Analogs

**Figure 5:**
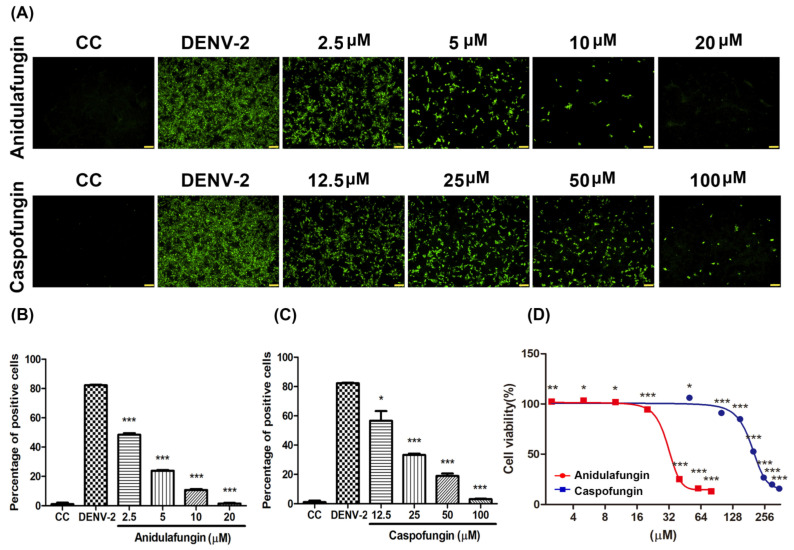
Anti-DENV-2 activity of anidulafungin and caspofungin, as determined using (**A**) IFA; (**B**,**C**) and ImageJ software. (**D**) Cell viability of anidulafungin and caspofungin. All data were obtained from at least three independent experiments, where significance was indicated as follows: * *p* < 0.05; ** *p* < 0.01; *** *p* < 0.001. Scale bar: 100 μM.

The structures of caspofungin and anidulafungin are similar to micafungin, containing a hexapeptide ring with an N-acyl side chain. Here, we investigated the anti-DENV-2 ability of two micafungin analogs, anidulafungin and caspofungin. Vero cells were infected with DENV-2 at MOI = 1 simultaneously with anidulafungin or caspofungin at indicated dosages. Following incubation at 37 °C for 2 days, DENV-2 infection was characterized using IFA (Figure 5A) and quantified using ImageJ software (Figure 5B,C). Anidulafungin and caspofungin both presented good antiviral activity against DENV-2 infection under the indicated dosages. The half-maximal inhibitory concentration (IC_50_) value of anidulafungin was 3.24 μM and the IC_50_ value of caspofungin was 20.78 μM. Neither compound affected cell viability at any of the tested concentrations (Figure 5D).

### 2.5. Antiviral Activity of Micafungin against Other Serotypes of DENV

**Figure 6:**
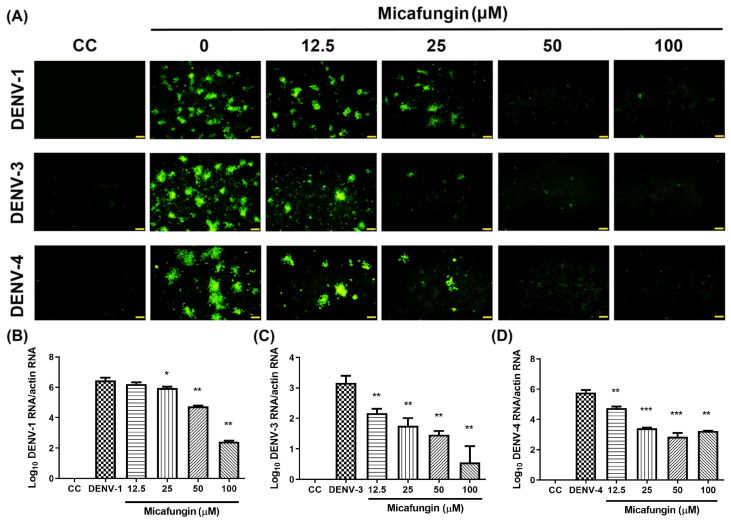
Antiviral effects of micafungin against other serotypes of DENV: (**A**) IFA results obtained under DENV-1, DENV-3, and DENV-4 infection. RNA levels of (**B**) DENV-1, (**C**) DENV-3, and (**D**) DENV-4, as determined using RT-qPCR assay. All data were obtained from at least three independent experiments, where significance was indicated as follows: * *p* < 0.05; ** *p* < 0.01; *** *p* < 0.001. Scale bar: 100 μM.

We also evaluated the effects of micafungin against other types of dengue virus (DENV-1, DENV-3, and DENV-4). Again, Vero cells were infected with DENV at MOI = 0.1 simultaneously with micafungin at various dosages. Following incubation at 37 °C for the 3 days, the outcomes were assessed using IFA (Figure 6A). Micafungin was shown to significantly inhibit all three types of DENV infection in a dose-dependent manner (12.5–100 μM). We also evaluated viral RNA levels via RT-qPCR assay using specific primers (Figure 6B–D). All three serotypes of micafungin were shown to reduce viral RNA levels.

## 3. Discussion

Micafungin is semi-synthesized from FR901379, a natural product of the fungus *Coleophama empedri*, which was discovered in 1989. FR901379 is a member of the echinocandin family with a hexapeptide with a long N-acyl side chain. It has been shown to fight Candida by inhibiting the synthesis of β-1,3-d-glucan in the walls of fungal cells. Sulfate residue on the surface enables water solubility superior to that of other echinocandins. Note however that FR901379 presents hemolytic activity associated with the side chain and poor oral absorption due to its high molecular weight. Micafungin has been synthesized with a modified N-acyl side chain with the aim of reducing hemolytic activity [14]. Micafungin presents linear kinetics after IV administration, and very few drug interactions have been reported [15,16]. In 2016, Kim et al. reported that micafungin has the ability to suppress EV71 infection [11]. In subsequent research, we demonstrated that micafungin can inhibit CHIKV infection by hindering viral replication, release, and stability [12]. Note that CHIKV and DENV are both arboviruses; however, they do not belong to the same family. In this study, we first confirmed that micafungin (6.25–100 µM) is able to suppress DENV-2 infection, viral RNA production, NS3 protein expression, and progeny yield (Figure 1A–D), with an IC_50_ value of 10.23 µM (see Figure 1A). Time-of-addition assays verified the inhibitory effects of micafungin in pre-treated, co-treated, and full-treatment groups. The IFA data showed that the infection rate of pre-treatment group decrease about 0.77-fold (Figure 2C), while the viral RNA level and viral title showed based 10 logarithms which reduced 0.89 logs (0.88-fold, Figure 2D) and 0.51 logs (0.71 fold, Figure 2E). The results of both show that the reduction level was very close. Additionally, the IFA data revealed that the post-treatment of micafungin possessed a minor decrease, but it was not observed at the RT-qPCR and TCID_50_ assay. We further analyzed the effect of micafungin after DENV-2 infection 1 h. Vero cells were inoculated with DENV-2 at MOI = 0.1 for an hour. After the inoculation was moved out, the Vero cells were treated with the indicated concentration of micafungin for 2 days incubation. Finally, the total RNA was extracted and analyzed by RT-qPCR. Micafungin possessed the inhibition at the concentration of 100 µM (Figure S1). Thus, micafungin at the higher concentration might inhibit the replication of DENV-2.

Viral binding and entry assays also demonstrated the effectiveness of micafungin in the early stage of DENV-2 infection. Viral binding is the initial processing of viral entry. A similar inhibition level of bind assay and entry assay might imply that the main effect of micafungin was to influence viral binding (Figure 3A,B). However, the anti-DENV activity of micafungin can be attributed to its effect on the envelope protein. Virucidal assays also revealed that micafungin had a direct influence on DENV-2 following incubation (Figure 3C–E). Note that envelope proteins of DENV are strongly involved in virus binding, entry, and stability. Thus, if it were possible for the compound to dock with the envelope protein, it might be possible to prevent virus entry into the cell. Molecular docking tools (Patchdock) confirmed that micafungin bound with the envelope protein of DENV (Figure 4A,B), that might block a conformational change during the viral entry. For micafungin, there are 4 hydrogen bonds that act on the chain B 269E (1), chain B 271Q (1), chain B 280T (2), and 9 covalent bonds that act on the chain A 244H, chain B 26E, chain B 27H, chain B 28G, chain B 202K, chain B 203D, chain B 204K, chain B 271Q, chain B 282H, respectively. Following the above findings, the possible mechanism of micafungin might be due to disrupt DENV-2 binding and destroy virion infectivity, via binding into the envelope proteins. In addition, anidulafungin and caspofungin, the other two analogs of micafungin, revealed the inhibition of DENV-2 infection (Figure 5A–C), but anidulafungin and caspofungin only showed the virucidal ability at the highest concentration (Figure S2). The data indicated that both micafungin analogs possessed the virucidal ability of DENV-2 only at high concentrations. Therefore, the main anti-viral effect of echinocandins might not only due to the virucidal ability. Other mechanisms might also involve the anti-DENV ability of these two analogs such as influencing viral binding. Moreover, the Cell Counting Kit-8 (CCK-8) assay results showed that there is not any cytotoxicity under micafungin 200 µM, Anidulafungin 20 µM, and caspofungin 200 µM which compared with cell control (Figure S3).

The envelope protein contains three ectodomains (Domain I~III), which play important roles in viral binding and entry. Previous studies have found that compounds are capable of binding to β-N-octylglucoside (β-OG) binding sites on the DENV envelope protein which could prevent the infection in the early stages of the disease [17,18]. Furthermore, the previous study indicated that the E-Dimer-dependent epitope (EDE), a conserved region of the four serotypes of DENVs, could also be a target to inhibit DENV infection [19]. Previous studies have reported on the anti-CHIKV ability of Micafungin [12]. Unlike, DENV, CHIKV belongs to the Togaviridae family; however, they are both arboviruses with similar transmission vectors, including Aedes aegypti and Aedes albopictus [20,21]. Furthermore, the envelope proteins associated with CHIKV and DENVs are both classified as class II fusion proteins [22], which suggests that they share similar infection processes. Nonetheless, the effects of micafungin against CHIKV infection occurred mainly in later stages, unlike the current study in which the effects of micafungin manifest in replication, release, and cell-to-cell transmission. Note that micafungin disrupted the stability of CHIKV as well as DENV. Researchers have also reported that micafungin inhibits the respiratory RNA virus, EV71, in the early stage of infection. Taken together, this evidence suggests that there are different mechanisms at work when applied to different viral infections.

In our study, we used micafungin at the concentration of 50 or 100 µM in Vero cells for 72 h without any further administration. However, the data at the cell level could not directly apply to clinical use, but we try to mimic the concentration used in the clinic roughly. According to the clinical pharmacokinetic data of micafungin in healthy adults, the volume distribution (Vd) is 0.2 L/kg, and the elimination half-life (t_1/2_) is 15.4h [23]. Clinically, micafungin was administered via IV injection once daily (QD), and the maximum dosage used is 8 mg/kg/day, which is equal to 520 mg/day for a 65 kg adult. Given that a 65 kg adult receives a daily dose of micafungin at 520 mg, the calculated plasma concentration is as follows. After the first dose administration, the plasma concentration is 40 µg/mL (31.48 µM), and the plasma concentration is 13.58 µg/mL (10.89 µM) before the second dose. After multiple administrations (about 3 dosages), the range of plasma concentration is between 20.57 µg/mL (16.18 µM) and 60.57 µg/mL (47.68 µM), while the average plasma concentration is 37.037 µg/mL (29.15 µM). All the plasma concentrations above are more than IC_50_ (10.23 µM). If the average plasma concentration is required as 50 µM, the daily dosage should be 891.75 mg, which is much higher than the dosage used clinically. However, a case report mentioned that the administration of 1400 mg at a single dose was safe to use (a total of seven 1400 mg infusions once every 2 weeks) [24]. In conclusion, a daily dose of 8 mg/kg is suggested. Although the safety of a single dose of micafungin at 1400 mg was reported in a case report, it still needs more evidence to illustrate the safety profile of micafungin. The working concentration of micafungin in this study is calculated based on the cell model, which cannot fully represent the actual working concentration in human bodies. Besides, the parameters used to calculate the concentration were obtained from healthy adults. There is no pharmacokinetics data of micafungin in dengue patients.

To summarize, DENV-2 infection is a complex process. In vivo, DENV-2 might not infect all cells of the body at first, but it is generally considered that the symptoms would become more severe with the rising number of infected cells. Those drugs that affect viral binding or entry possess the ability to stop DENV-2 infection. That might help to reduce the secondary infection to prevent the exacerbation of the disease. Note that the current study was limited to the application of micafungin to DENV infection in vitro. Gaining a realistic appraisal of the value of micafungin as an antiviral drug will require in vivo analysis.

## 4. Materials and Methods

### 4.1. Cells, Viruses, and Compounds

Vero cells (ATCC: CCL-81) were cultured in DMEM/high-glucose medium (Catalog number: SH30022.02; Hyclone, UT, USA) with 5% fetal bovine serum (FBS) (Catalog number: SH30088.03; Hyclone, UT, USA), 2.5% HEPES (Catalog number: 03-025-1B; Biological Industries, CT, USA), and antibiotics at 37 °C under 5% CO_2_. DENV-1 (Strain: US/Hawaii/1944), DENV-2 (Strain: Thailand/16681/1984), DENV-3 (Strain: Philippines/H87/1956), and DENV-4 (Strain: Philippines/H241/195) were propagated by Vero cells. Micafungin (CAS Number: 208538-73-2), anidulafungin (CAS Number: 166663-25-8), and caspofungin (CAS Number:179463-17-3) were purchased from Commercial company (Selleck Chemicals LLC, TX, USA) and dissolved as stocks for experiments.

### 4.2. Viral Quantification and Immunofluorescence Assay (IFA)

Virus quantification began with ten-fold serial dilution of viral supernatant. The dilute solution was added to Vero cells for 1 h absorption and then replaced with 1.5% methylcellulose for 2-day incubation. Viral titers were calculated as fluorescent focus units (FFU) per ml. TCID_50_ assays were performed by adding the above-mentioned dilutions to Vero cells in 96-well plates. Each dilution was repeated 6 times. Following incubation for 4–5 days, DENV-induced cytopathic effect was observed under microscope.

IFA involved incubating virus cells with the primary antibody and then the secondary antibody at room temperature following fixation. PBS washing was performed three times between each step. Images were captured using an inverted OLYMPUS IX73 fluorescence microscope (Olympus, Tokyo, Japan). Anti-DENV NS3 protein antibodies (Catalog number: GTX124252; 1:400 dilution; GeneTex, Irvine, CA, USA) and anti-rabbit DL488 (Catalog number: GTX213110-04; 1:1000 dilution; GeneTex, Irvine, CA, USA) were used for IFA. Anti-Flaviviridae envelope antibodies (Homemade; 1:400 dilution) and anti-mouse DyLight488 (Catalog number: GTX213111-04; 1:1000 dilution; GeneTex, Irvine, CA, USA) were used for FFU. DAPI was used to stain the cell nucleus.

### 4.3. RNA Isolation and RT-qPCR

Total RNA was extracted from the tested cells using RNA extraction reagent (Catalog number: MRE-3200; EBL, New Taipei City, Taiwan). RNA expression was quantified using the QuantiTect SYBR Green RT-PCR Kit (Catalog number: 204243; Qiagen, Hilden, Germany). The primers were adopted from a previous study [13,25]. A Mic qPCR Cycler (Bio Molecular Systems, Queensland, Australia) was implemented under the following program: 50 °C for 30 min and 95 °C for 15 min followed by 40 cycles of 95 °C for 20 s, 55 °C for 30 s, and 72 °C for 30 s. The forward and reverse primers are listed in Supplementary Table S1 with actin serving as an internal control.

### 4.4. Western Blot Analysis

Total proteins in the tested cells were lysed using 1X Radioimmunoprecipitation Assay (RIPA) lysis buffer (Catalog number: ab156034; Abacam, Cambridge, MA, USA) and separated using 10% sodium dodecyl sulfate polyacrylamide gel electrophoresis (SDS-PAGE gel). The proteins were then transferred to polyvinylidene difluoride (PVDF) membranes for incubation with a 1:1000 dilution of anti-DENV NS3 primary antibodies or GAPDH antibodies (Catalog number: GTX100118; 1:5000 dilution; GeneTex, Irvine, CA, USA). After washing out those antibodies, Goat anti-rabbit IgG H&L (HRP) (Catalog number: ab6721; 1:1000 dilution; Abacam, Cambridge, MA, USA) was added. Chemiluminescent detection was performed using the Immobilon^®^ Western chemiluminescent horseradish peroxidase (HRP) substrate (Catalog number: P90720; Millipore, Billerica, MA, USA) in accordance with the manufacturer’s protocol. The UVP Auto Chemi Image system, MultiGel-21, (TOP BIO, New Taipei City, Taiwan) was used to capture and process images.

### 4.5. Virucidal Assay

The indicated concentrations of test drugs were added into DENV-2 stock (about 2 × 10^6^ pfu/mL) for 1 h incubation at 37 °C. Then, the incubated DENV-2 stock was diluted 100-fold (MOI = 0.1) and 1000-fold (MOI = 0.01) to exclude the effect of test drugs. The DENV-2 stock diluent was added into Vero cells at 24-well plates for 1 h absorption at 37 °C. Subsequently, the diluent was removed, and the infected cells were covered with DMEM containing 1.5% (*w*/*v*) methylcellulose and 2% FBS. After 2 days of incubation, the infected cell was fixed and determined the virus infection by immunofluorescence assay. The quantitation was used ImageJ and foci counting.

### 4.6. Cell Viability and CCK-8 Assays

Vero cells were treated with the indicated concentrations of drugs for 2- or 3-days incubation. The cell viability assay was detected by crystal violet assay and CCK-8 assay. Crystal violet assay is following treated cells fixed using a mixture of acetone and methanol at 4 °C for 15 min incubation. The cells were stained by 0.1% crystal violet at room temperature for 5 min, and then washed three times. Cell viability of crystal violet assay was determined by (OD_570 treatment_/OD_570 CC_) × 100%, and the results were normalized using control cells (CCs). For CCK-8 assay, the 10% CCK-8 solution (Catalog number: 96992; Sigma-Aldrich, St. Louis, MO, USA) was added to the treated cells and incubated for 1 to 4 h. Cell viability of CCK-8 assay were determined by (OD_450 treatment_ − OD_650 treatment_/OD_450 CC_ − OD_650 CC_) × 100%, and the results were normalized using control cells (CCs).

### 4.7. Molecular Docking

Docking simulations were performed using PatchDock to predict the molecular docking between ligands and the DENV-2 envelope glycoprotein (PDB: 1OAN) [26]. The DENV-2 envelope protein structure was selected as the receptor molecule for docking. For ligand molecule, the chemical structures of micafungin, anidulafungin, and caspofungin were obtained from spiderchem, and the file format was transformed into PDB file using Pymol (PyMOL Molecular Graphics System, Version 2.4.1, Schrödinger, and LLC). Then, the molecular docking was performed on the PatchDock, which was a geometry-based molecular docking algorithm [27]. The type of protein-small ligand was selected for computing the docking program, and the outcome presented the top 20 solutions ranked by the geometry score. Finally, the optimal docking conformation was selected according to the highest dock score [27,28].

### 4.8. Statistical Analysis

The Student’s *t*-test was used to analyze data with a *p*-value of less than 0.05 considered significant [29]. All statistical analysis was conducted using Prism 8.0 software (GraphPad Software Inc., San Diego, CA, USA).

## 5. Conclusions

This study was the first report to describe the anti-DENV ability of micafungin. Micafungin could inhibit DENV-2 infection and the possible mechanism of micafungin might influence DENV-2 binding to block DENV-2 entry. Micafungin also revealed virucidal ability. The result of molecular docking indicated that micafungin could bind into the cavity between the envelope proteins dimer. The evidence helped to explain how micafungin inhibited at the early stage and reduced the virus infectivity. The other echinocandins showed the inhibition of DENV-2 infection which proved micafungin and its analogs might possess the same anti-DENV ability. Finally, micafungin was proved the anti-viral ability in the other three serotypes of DENVs. Thus, micafungin could be a candidate for broad anti-DENV treatment.

## Data Availability

Data are contained within the article.

## Supplementary Materials

The following are available online at https://www.mdpi.com/article/10.3390/ph14040338/s1, Figure S1: The effect of micafungin on DENV-2 replication, Figure S2: The virucidal assay of anidulafungin and caspofungin, Figure S3: The cell viability via CCK-8 assay, Table S1: RT-qPCR primers, Table S2: The structure, preparation, Cytotoxicity (CC_50_), inhibition concentration (IC_50_), and selective index (SI) of micafungin and its analogs.
