# Supplementary material for: Micafungin Inhibits Dengue Virus Infection through the Disruption of Virus Binding, Entry, and Stability
Source: Pharmaceuticals (Basel). 2021 Apr 7;14(4):338. doi: 10.3390/ph14040338 (PMC8067805; doi:10.3390/ph14040338)
Supplement: Supplementary file 1 [file pharmaceuticals-14-00338-s001.pdf]

Supplementary Table S1. RT-qPCR primers

<table>
<tr><th></th><th>Forward primer</th><th>Reverse primer</th></tr>
<tr><td>DENV-1</td><td rowspan="4">5′-CAATATGCTGAAACGCGAGAGAAA-3′</td><td>5′-CGCTCCATACATCTTGAATGAG-3′</td></tr>
<tr><td>DENV-2</td><td>5′-AAGACATTGATGGCTTTTGA-3′</td></tr>
<tr><td>DENV-3</td><td>5′-AAGACGTAAATAGCCCCCGAC-3′</td></tr>
<tr><td>DENV-4</td><td>5′-AGGACTCGCAAAAACGTGATGAAT-3′</td></tr>
<tr><td>Actin</td><td>5′ATTGCCGACAGGATGCAGAA-3′</td><td>5′-GCTGATCCACATCTGCTGGAA-3′</td></tr>
</table>

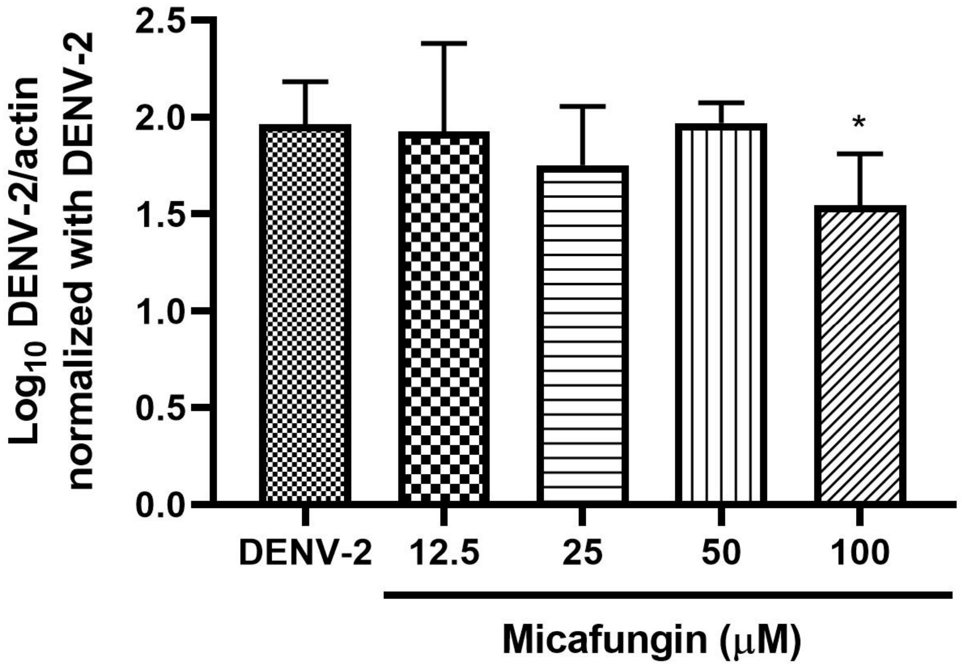

Figure S1. The effect of micafungin on DENV-2 replication. RT-qPCR assay was used to quantify the RNA level. All data were obtained from at least four independent experiments, where significance was indicated as follows: *p<0.05; **p<0.01; ***p<0.001.

(A)

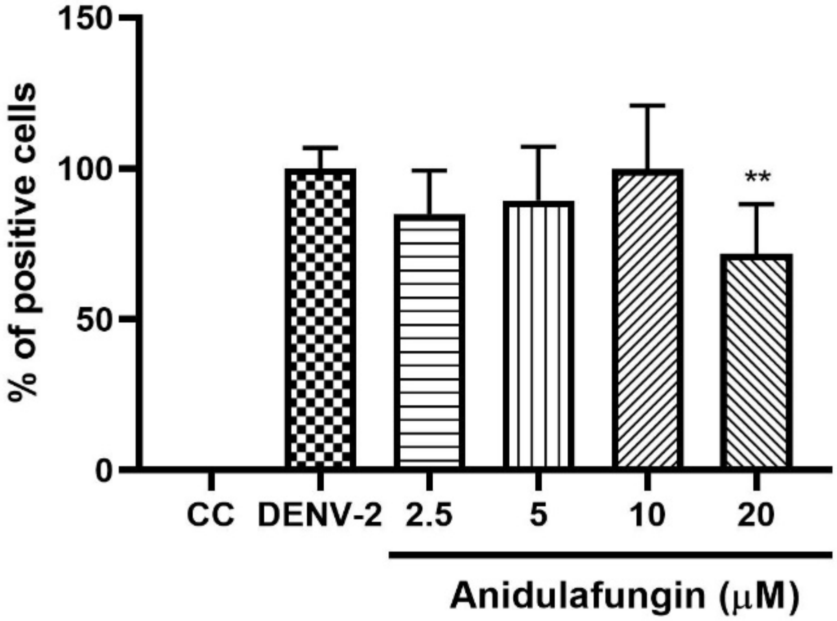

(B)

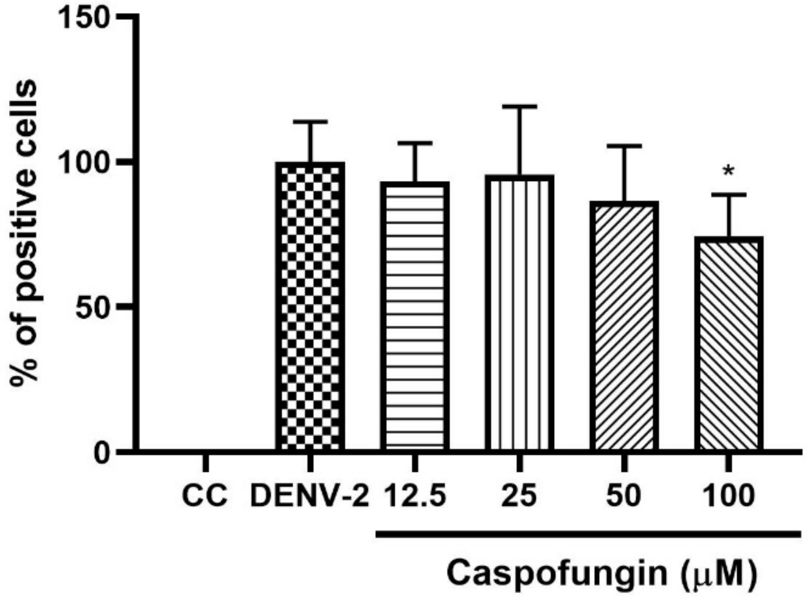

Figure S2. The virucidal assay of **(A)** anidulafungin and **(B)** caspofungin. IFA was used to calculate the foci of DENV-2, and the results were performed by percentage of positive cell compared with virus control. All data were obtained from at least six independent experiments, where significance was indicated as follows: *p<0.05; **p<0.01; ***p<0.001.

(A)

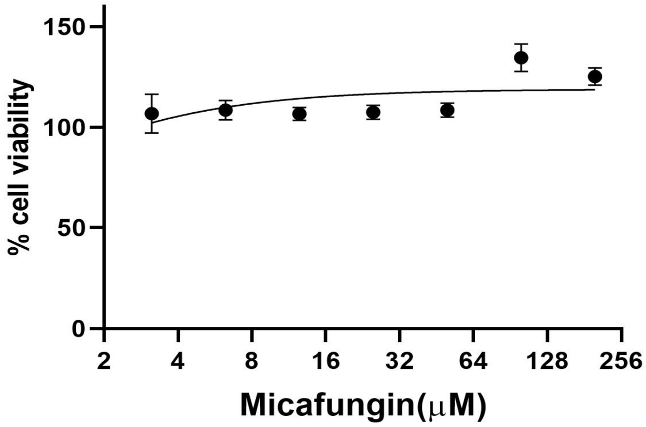

(B)

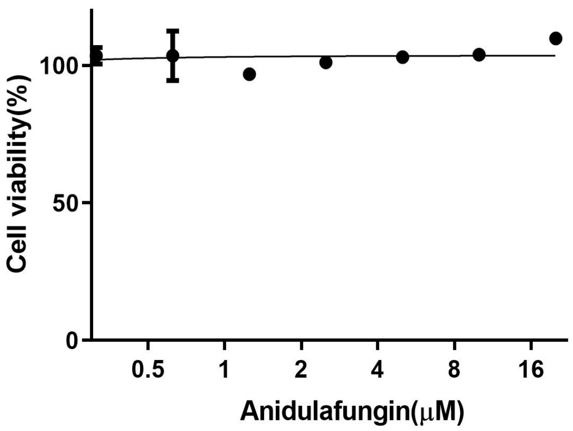

(C)

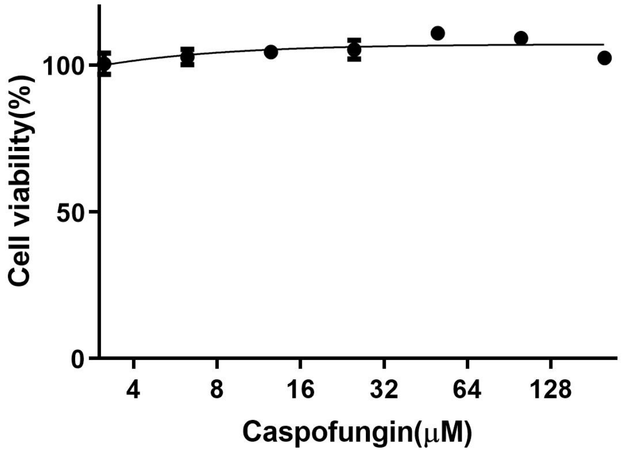

Figure S3. The cell viability via CCK-8 assay. The cell viability was determined by CCK-8 assay (A) micafungin for 3day incubation, (B) anidulafungin, and (C) caspofungin for 2day incubation.

Supplementary Table S2. The structures, preparations, Cytotoxicity (CC_{50}), inhibition concentration (IC_{50}), and selective index (SI) of micafungin and its analogs

Drug name	Structure	Stock concentration / Solvent	The highest concentration / Cytotoxicity	IC_{50}	CC_{50}	SI
Micafungin	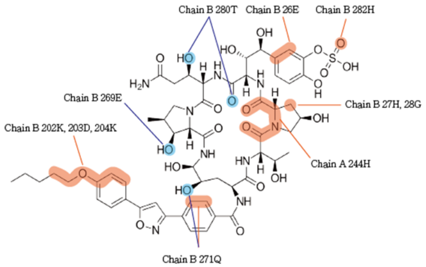	50mM / Water	100µM / none	10.23µM	>200µM	>19.55
Anidulafungin	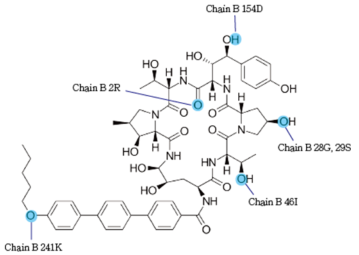	50mM / DMSO	20µM / none	3.24µM	>20µM	>6.17
Caspofungin	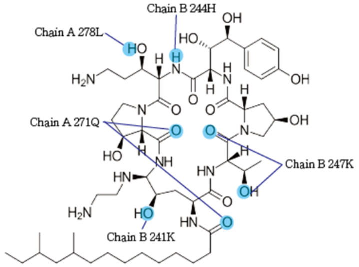	10mM / Water	100µM / none	20.78µM	>200µM	>9.62

*The hydrogen bond is shown in blue, and the covalent bond shown in orange.
